# Hydatidiform Mole

**Published:** 1908-11-14

**Authors:** 


### Obstetrics.

### HYDATIDIFORM MOLE.

A considerable amount of attention has been drawn in the last decade to this condition, on account of its undoubted relation to chorion epithelioma of the uterus, apart from the very serious effects it has upon the patient on its own account. It is, of course, a disease of the chorion, and one which occurs early in embryonic life before the placenta is completely formed. In all likelihood it is a degeneration which begins very early, probably as soon as the chorionic villi are well formed, though we have no *data* to show the exact time at which it may begin.

The chorionic villi are transformed into vesicles, oval in shape and varying in size, sometimes reaching an inch in diameter. These vesicles are arranged like beads for the most part, but the branching of the villi often produces clusters. In the early embryos thus affected, the whole chorion may be found degenerate, whilst the amnion still persists as a distinct cavity. In older specimens it is the rule for the amnion to be no longer recognisable. The embryo itself disappears very early as a rule, because it dies owing to its blood supply being cut off, and then undergoes dissolution. In a few reported cases the disease appears only in a part of the chorion, and may exist along with an otherwise well-formed placenta. In such a case the embryo may still exist.

It occasionally happens that in a twin pregnancy one embryo alone shows the disease, the other one being apparently normal. The size to which a hydatidiform mole may attain is of course very variable, but a weight of 5 lb. is stated to have been attained in some cases. Microscopically the disease is found to be limited to the chorionic villi, and shows itself in a two-fold manner. First, there is a marked proliferation of the trophoblast covering the villi, both layers of which can be seen to share in the change. The syncytium buds very actively and produces large thick irregular plasmodial masses with many nuclei and no cell divisions, whilst the Langhans layer proliferates and forms masses of the typical discrete cells with marked vesicular nuclei which characterise this structure. Secondly, the stroma of the villi degenerates, forming at first what appears to be a myxomatous tissue, and later this liquefies and all cell structure disappears. It is, however, not true myxomatous degeneration, for it is stated that the fluid produced contains no mucin. The formation, however, of so much fluid in the villous trunks gives rise to the vesicles from which the name of the condition is derived, and the pressure thus produced leads to thinning of the coverings, except at those parts where active proliferation is going on. We do not know the actual cause of the disease, but it is fairly clear that it must be a primary disease of the embryo and its coverings, and not a disease of the decidua or any other uterine tissue. There is no reason whatever to believe that syphilis, tubercle, or any other general disease of the mother plays any part in it. An interesting and significant feature has been brought to light recently—namely, the association of corpus luteum cysts in the ovary and general over-production of lutein tissue in that organ, with hydatidiform mole. A sufficient number of cases have now been described to show that this association is not an accidental circumstance. At present, however, we do not know enough about the corpus luteum to be able to state that over production of lutein tissue is the cause of hyperactivity of the trophoblast, although the experiments of Frankel and others go to show that there is some relation between the two.

Clinically, hydatidiform mole is often very difficult to distinguish from a normal pregnancy. Two features, however, stand out as important aids to diagnosis—namely, first an excessive development and size of the uterus for the period of amenorrhœa, and secondly the early appearance of a blood-stained discharge with occasional actual hæmorrhage. The first sign is so marked that with a known period of three months amenorrhœa, the uterus may attain the size of a five-months' pregnancy. For shorter periods of amenorrhœa the size of the uterus is correspondingly less, but, of course, the disproportion is less obvious. The discharge is often described as like " red currant juice," but is not in any way characteristic unless a vesicle is discharged with it. It is important to note, however, that the patient may become seriously anæmic from the discharge and hæmorrhage which may occur. The uterus containing a mole may feel " boggy " instead of elastic, and a sign of some importance is the occurrence of irregular uterine contractions occurring only in limited areas of the uterus. The disappearance of the embryo at an early stage leads to the retrocession of the breast signs and non-appearance of other signs of a progressive pregnancy. As a general rule, after a careful consideration of the history and the above-mentioned signs, it will not often be possible to do more than suspect a hydatid mole unless vesicles have been discharged or the os uteri is sufficiently open to permit the finger to feel the vesicles.

Some of these hydatidiform moles are expelled spontaneously, whilst in others the condition of the patient is such as to call for exploration of the interior of the uterus and subsequent evacuation of the mole. When evacuation has been determined upon, the cervix should first be dilated with aseptic tents or metal dilators. The former nearly always set up uterine contractions, but these are often unable to empty the uterus, and the final evacuation must be carried out with the fingers. Two fingers in the uterus can usually manage this, in spite of the apparent size of the organ. Pressure from without enables successive parts to be brought in reach and the vesicles gradually detached from the uterine wall. Care is very necessary here, for the trophoblastic proliferation leads to invasion of the uterine wall by the villi, and even perforation of the wall by them has been recorded. If this operation has been carried out in an aseptic manner the uterus speedily involutes. The great danger is that some portion in the uterine wall may lead to the formation of chorion epithelioma instead of undergoing atrophy.

